# Polymer Having Dicationic Structure in Dumbbell Shape for Forward Osmosis Process

**DOI:** 10.3390/polym11030571

**Published:** 2019-03-26

**Authors:** Taehyung Kim, Changha Ju, Chanhyuk Park, Hyo Kang

**Affiliations:** Department of Chemical Engineering, Dong-A University, 37 Nakdong-Daero 550beon-gil, Saha-gu, Busan 49315, Korea; xogud1290@donga.ac.kr (T.K.); 1771088@donga.ac.kr (C.J.); chan941121@donga.ac.kr (C.P.)

**Keywords:** draw solute, dicationic structure, forward osmosis, lower critical solution temperature (LCST), recovery

## Abstract

The thermal-responsive polymers, poly(alkane-1,#-diylbis(tri-*n*-butylphosphonium) 4-vinylbenzenesulfonate) (PSSBP#, # = 8, 6, and 4), where # is the number of carbon atoms in the central bridge structure of the dicationic phosphonium moiety, were synthesized to examine their potential application as draw solutes in forward osmosis (FO). The polymers exhibited low critical solution temperature (LCST) characteristics in aqueous solutions, which is essential for recovering a draw solute from pure water. The LCSTs of the 20 wt% aqueous solutions of PSSBP8, PSSBP6, and PSSBP4 were confirmed to be approximately 30, 38, and 26 °C, respectively, which is advantageous in terms of energy requirements for the recovering draw solute. When the concentration of the PSSBP4 draw solution was 20 wt%, water flux and reverse solute flux were approximately 1.61 LMH and 0.91 gMH, respectively, in the active layer facing the draw solution (AL-DS) system when the feed solution was distilled water. The PSSBP# thermal-responsive draw solute has considerable potential for use as a next-generation draw solute because of its excellent osmotic performance and efficient recovery. Therefore, this study provides inspiration for novel ideas regarding structural transformations of polymers and their applicability as draw solutes.

## 1. Introduction

**Figure 1:**
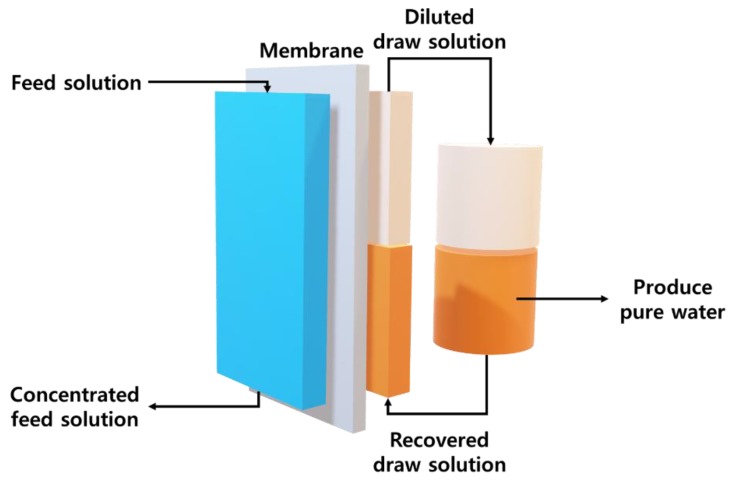
Schematic diagram of forward osmosis (FO) process.

Water scarcity is a critical problem facing humanity, as it is essential to supply clean water to societies worldwide. To date, technologies have been developed to obtain pure water from saline, waste, and agricultural water mainly in the form of water separation technologies, which can be classified as distillation or membrane methods [1]. The distillation method separates pure water from wastewater using selective boiling point properties [2]. This method can result in perfect separation of pure water from impurities. Membrane methods use a selective barrier to block unwanted molecules or ions and allow the desired substances, such as water molecules, to pass through. Membrane methods can be subdivided into reverse osmosis (RO) and forward osmosis (FO) processes [3]. Distillation and RO require significant energy inputs in the form of heat for vaporization and mechanical pressure to produce pure water. However, FO uses a small amount of energy because it takes advantage of the natural osmosis phenomenon. The osmosis phenomenon in the FO process is caused by the osmotic gradient between the feed (e.g., saline water) and the draw solution. Moreover, the draw solution (a draw solute dissolved in aqueous solution) must exhibit an osmotic potential higher than that of the feed to allow for the production of pure water [4,5]. As a final outcome, FO technology can achieve higher energy efficiency than other technologies for water purification and has attracted significant attention as a next-generation technology due to its low fouling potential, low energy consumption, simplicity, and reliability [6]. FO is a membrane process that produces water in substantial quantities using a draw solute and recovery process that separates the draw solute by application of an external stimulus, which can allow for the recycling of the draw solute (Figure 1) [3,5]. For further development of FO, energy and cost reduction must be achieved during the recovery and reuse of draw solutes [7].

External-stimulus-responsive draw solutes have been previously developed, which is summarized in Table 1. External-stimulus-responsive draw solutes can be divided into a chemical-stimulus-responsive and physical-stimulus-responsive method depending on the type of stimulation applied. The chemical-stimulus-responsive method refers to a method of reusing the draw solute by adding a gas or salt and adjusting pH. This method can be subdivided largely into carbon-dioxide-responsive, salt-responsive, and pH-responsive methods. Carbon-dioxide-responsive draw solutes exhibit osmotic pressure by utilizing the ammonium cation and bicarbonate anion formed by chemical reaction between the amine group, carbon dioxide, and water [8,9]. For example, Elimelech et al. investigated an ammonium bicarbonate (NH_4_HCO_3_) draw solute derived from ammonia (NH_3_) and carbon dioxide (CO_2_) in the aqueous phase [8]. Although this draw solute achieves a high osmotic gradient, decomposition of NH_4_HCO_3_ to form NH_3_ and CO_2_ in the recovery process requires a large amount of thermal energy, and the quality of obtained water is poor due to residual draw solutes [10]. Salt-responsive draw solutes are recovered using a sedimentation method that relies on physicochemical bonding with metal cations [11,12,13,14,15]. Among the salt-responsive draw solutes developed to date, the green-material-based and multivalent draw solute potassium tannate, aluminum sulfate, and magnesium sulfate have been applied in FO processes [13,14,15]. pH-Responsive method signifies a method in which recovery characteristic of draw solutes are expressed in accordance with acid/base change—a pH change—of the aqueous draw solution. Cai et al. introduced poly[2-(dimethylamino)ethyl methacrylate] (PDMAEMA) as a polymeric draw solute for the FO process, and its recovery property was performed by adjusting the pH of an aqueous solution [16].

The physical-stimulus-responsive method can also be used to recover draw solutes using an applied physical external force. This method can be subdivided into magnetic-responsive, electro-responsive, light-responsive, and thermal-responsive recovery strategies. Magnetic-responsive draw solutes are typically organic-inorganic complexes formed using a material with an osmotic gradient (i.e., polyacrylic acid or poly(ethylene glycol)diacid) on the surface of a magnetic nanoparticle (MNP) that is sensitive to magnetic fields [17,18,19,20,21]. Chung et al. developed a magnetic-responsive draw solute with a core-shell structure consisting of an MNP in the core and polyacrylic acid in the shell to induce an osmotic gradient [18]. However, due to the nature of the MNP, nanoparticle agglomeration occurred, and the particle size increased with the number of repetitive magnetic recovery processes, resulting in reduction of FO performance, e.g., water flux [22]. Zhang et al. studied actively electro-responsive polymeric hydrogel for forward osmosis processes [23,24]. Among them, hyaluronic acid and poly(vinyl alcohol) (HA/PVA) hydrogels have been applied as electro-responsive draw solutes where water is produced by applying voltage to both the feed solution and the HA/PVA hydrogel [23]. Zhang et al. attached the HA/PVA hydrogel to one side of a semi-permeable membrane used in FO, feed solution was supplied to other side, and pure water was obtained by applying a voltage to both the feed solution and the hydrogel.

Light-responsive draw solute refers to the solute that possesses the responsiveness to light of a specific wavelength, such as visible or ultraviolet rays, to exhibit recovery property. Lu et al. studied light-responsive and heterodimer-type draw solute, which is composed of Ag and Fe_3_O_4_ nanoparticles with coupled [25]. When heterodimeric draw solute is illuminated with solar ray, Ag nanoparticles absorb the ray centered at a wavelength of 420 nm due to plasmonic effect. Based on this effect, the physicochemical interaction between dimers occurs, which can lead to cohesion for draw solute recovery, and consequently the light-responsive draw solute can be recovered.

Thermal-responsive draw solutes utilize homogeneous changes in the heterogeneous phase or heterogeneous changes in homogeneous phases in aqueous solutions before or after a certain critical temperature—either the lower critical solution temperature (LCST) or the upper critical solution temperature (UCST) [26,27,28,29,30]. The LCST is the critical temperature below which the component of a solution is immiscible, and the UCST functions in the opposite manner. In a previous report, a copolymer composed of *N*-isopropylacrylamide (NIPAM), which exhibits LCST properties for draw solute recovery and contains an ionic moiety for obtaining pure water, was used as a draw solute [29,30]. However, since the recovery and drawing functions are independently controlled in the copolymeric draw solute, a trade-off relationship was formed. If the proportion of the drawing part contained on the copolymeric draw solute is lower than that of the recovery part, the osmotic pressure will be low, and conversely, the recovery of the draw solute will be poor. Therefore, a homopolymer structure composed of a monomer capable of simultaneous recovery and drawing water is advantageous in terms of FO performance. Additionally, it is important to regulate the recovery temperature of the thermal-responsive draw solute exhibiting LCST or UCST characteristics to be similar to that of the FO operating temperature for improved energy efficiency during draw solute recovery.

In this study, we synthesized a homopolymer series with dumbbell-shaped dicationic structures, which are well-known to exhibit temperature responsiveness, as a draw solute for FO. The potential of this homopolymer series with LCST characteristics as a next-generation draw solute for FO was systematically investigated, evaluating the relevant FO performance and recovery properties.

## 2. Materials and Methods

### 2.1. Materials and Instrumentation

Tributylphosphine and sodium 4-vinylbenzenesulfonate were purchased from Sigma Aldrich Co., LLC. (St. Louis, MO, USA), and 1,4-dibromobutane, 1,6-dibromohexane, 1,8-dibromooctane, and tetrabutylphosphonium bromide were purchased from Tokyo Chemical Industry Co., Ltd. (Japan). 2,2′-Azoisobutyronitrile (AIBN), used as an initiator for polymerization, was purified by crystallization using methanol. *N*,*N*’-Dimethylformamide (DMF) was dried over molecular sieves (4 Å), and pure diethyl ether was obtained by distillation after adding calcium chloride to unpurified diethyl etherl; it was then treated with metallic sodium and distillated. All other reagents and solvents were used without further purification.

To identify the molecular structure of the substances, proton nuclear magnetic resonance (^1^H-NMR; Agilent, Santa Clara, CA, USA, MR400 DD2) was performed at 400 MHz. The rheological properties of the samples were investigated by measuring shear rate changes using a cone-and-plate rheometer with a diameter of 40 mm and a cone angle of 2° (TA Instrument, New Castle, DE, USA, AR G2). Conductivity was measured using a conductivity meter (METTLER TOLEDO, Columbus, OH, USA, Seven2Go pro). The osmotic pressure was measured using an osmometer (KNAUER, Berlin, Germany, SEMI-MICRO OSMOMETER K-7400) via the cryoscopic method. To determine the LCST of the prepared polymers, a UV-Vis spectrophotometer (EMCLAB Instruments GmbH, Duisburg, Germany, EMC-11D-V Spectrophotometer) equipped with a temperature controller (Misung Scientific Co.， Ltd., Yangju, Korea, TC200P) was used. The water and reverse solute fluxes were determined by measuring the height difference between the solution levels on both sides of a custom-made U-shaped tube and the conductivity difference before and after the experiment, respectively.

### 2.2. Preparation of Alkane-1,#-diylbis(tri-n-butylphosphonium) Bromide (BP#Br_2_)

Dumbbell-shaped dicationic phosphonium bromide, alkane-1,#-diylbis(tri-*n*-butylphosphonium) bromide (BP#Br_2_), where # is the number of carbons in the central bridge structure of the dicationic phosphonium moiety, was generated using a bimolecular nucleophilic substitution reaction (SN_2_ reaction). The BP#Br_2_ samples were obtained using a previously reported procedure [33]. For example, the synthetic conditions of BP8Br_2_ were as follows: 8.93 g (44.11 mmol) of tributylphosphine and 4.00 g (14.71 mmol) of 1,8-dibromooctane were dissolved in acetone (20 mL). The temperature was maintained at 45 °C, and the solution was reacted under magnetic stirring under a nitrogen atmosphere for 60 h. The material to be obtained from the solution was precipitated using diethyl ether and separated by filtration. Subsequently, the purity of BP8Br_2_ was increased using the Soxhlet process with diethyl ether to remove impurities. The remaining solvent in the obtained material was dried under vacuum overnight to obtain BP8Br_2_. A ^1^H-NMR spectrometer was used to characterize the BP8Br_2_ structure and composition (see Supplementary Materials Figure S1).

^1^H-NMR of BP8Br_2_ [400 MHz, D_2_O, *δ*/ppm]: 0.88–0.97 (m, 18H, (C*H*_3_–CH_2_–)), 1.32–1.63 (m, 36H, (–C*H*_2_–C*H*_2_–CH_2_–P^+^–), (P^+^–CH_2_– (C*H*_2_)_6_–CH_2_–P^+^)), 2.11–2.23 (m, 16H, (–C*H*_2_–P^+^–)).

Other members of the BP#Br_2_ series, such as BP6Br_2_ and BP4Br_2_, were synthesized using the same procedure as that used for preparation of BP8Br_2_, except differing amounts of 1,6-dibromohexane and 1,4-dibromobutane were used. For instance, BP6Br_2_ and BP4Br_2_ were prepared using 1,6-dibromohexane (3.59 g, 14.71 mmol) and 1,4-dibromobutane (3.18 g, 14.71 mmol), respectively. The structures of BP6Br_2_ and BP4Br_2_ were confirmed via ^1^H-NMR spectroscopy (Figures S2 and S3).

^1^H-NMR of BP6Br_2_ [400 MHz, D_2_O, *δ*/ppm]: 0.90–0.98 (m, 18H, (C*H*_3_–CH_2_–)), 1.42–1.65 (m, 32H, (–C*H*_2_–C*H*_2_–CH_2_–P^+^–), (P^+^–CH_2_–(C*H*_2_)_4_–CH_2_–P^+^)), 2.13–2.26 (m, 16H, (–C*H*_2_–P^+^–)).

^1^H-NMR of BP4Br_2_ [400 MHz, D_2_O, *δ*/ppm]: 0.91–0.98 (m, 18H, (C*H*_3_-CH_2_–)), 1.41–1.81 (m, 28H, (–C*H*_2_–C*H*_2_–CH_2_–P^+^–), (P^+^–CH_2_–(C*H*_2_)_2_–CH_2_–P^+^)), 2.17–2.34 (m, 16H, (–C*H*_2_–P^+^–)).

### 2.3. Preparation of Alkane-1,#-diylbis(tri-n-butylphosphonium) 4-vinylbenzenesulfonate (SSBP#) and Tetrabutylphosphonium 4-vinylbenzenesulfonate (SSMP)

The alkane-1,#-diylbis(tri-*n*-butylphosphonium) 4-vinylbenzenesulfonate (SSBP#) series and tetrabutylphosphonium 4-vinylbenzenesulfonate (SSMP) were prepared according to a previously reported method [34]. The SSBP8 was synthesized using an extraction technique with dichloromethane and distilled water. Sodium 4-vinylbenzenesulfonate (6.76 g, 32.78 mmol) and BP8Br_2_ (11.07 g, 16.39 mmol) were first dissolved in distilled water (40 mL) in a 100 mL beaker and magnetically stirred at room temperature for 1 h. The product was extracted three times using dichloromethane and concentrated using a rotary evaporator to obtain viscous yellowish liquid (Figure S4).

^1^H-NMR of SSBP8 [400 MHz, D_2_O, *δ*/ppm]: 0.86–0.92 (m, 18H, (C*H*_3_–CH_2_–)), 1.26–1.55 (m, 36H, (CH_3_–C*H*_2_–C*H*_2_–CH_2_–P^+^–), (P^+^–CH_2_–(C*H*_2_)_6_–CH_2_–P^+^)), 2.04–2.15 (m, 16H, (–C*H*_2_–P^+^–)), 5.38–5.43 (dd, 2H, (C*H*_2_=CH–Ph–)), 5.90–5.96 (dd, 2H, (C*H*_2_=CH–Ph–)), 6.77–6.86 (dd, 2H, (CH_2_=C*H*–Ph–)), 7.57–7.77 (dd, 8H, (CH_2_=CH–*Ph*–SO_3_^−^)).

Other members of the SSBP# series, i.e., SSBP6 and SSBP4, and SSMP were synthesized using the same procedure applied for the preparation of SSBP8, except different amounts of BP6Br_2_, BP4Br_2_, and tetrabutylphosphonium bromide were used. For example, SSBP6, SSBP4, and SSMP were prepared using BP6Br_2_ (10.61 g, 16.39 mmol), BP4Br_2_ (10.16 g, 16.39 mmol), and tetrabutylphosphonium bromide (11.12 g, 32.78 mmol), respectively. The structures of each monomer are shown in Figures S5–S7.

^1^H-NMR of SSBP6 [400 MHz, D_2_O, *δ*/ppm]: 0.86–0.94 (m, 18H, (C*H*_3_–CH_2_–)), 1.35–1.53 (m, 32H, (–C*H*_2_–C*H*_2_–CH_2_–P^+^–), (P^+^–CH_2_–(C*H*_2_)_4_–CH_2_–P^+^)), 1.99–2.11 (m, 16H, (–C*H*_2_–P^+^–)), 5.37–5.43 (dd, 2H, (C*H*_2_=CH–Ph–)), 5.87–5.96 (dd, 2H, (C*H*_2_=CH–Ph–)), 6.74–6.84 (dd, 2H, (CH_2_=C*H*–Ph–)), 7.54–7.79 (dd, 8H, (CH_2_=CH–*Ph*–SO_3_^−^)).

^1^H-NMR of SSBP4 [400 MHz, D_2_O, *δ*/ppm]: 0.83–0.93 (m, 18H, (C*H*_3_–CH_2_–)), 1.30–1.64 (m, 28H, (–C*H*_2_–C*H*_2_–CH_2_–P^+^–), (P^+^–CH_2_– (C*H*_2_)_2_–CH_2_–P^+^)), 2.10–2.31 (m, 16H, (–C*H*_2_–P^+^–)), 5.22–5.29 (dd, 2H, (C*H*_2_=CH–Ph–)), 5.78–5.86 (dd, 2H, (C*H*_2_=CH–Ph–)), 6.66–6.76 (dd, 2H, (CH_2_=C*H*–Ph–)), 7.37–7.58 (dd, 8H, (CH_2_=CH–*Ph*–SO_3_^−^)).

^1^H-NMR of SSMP [400 MHz, D_2_O, *δ*/ppm]: 0.87–0.95 (m, 12H, (C*H*_3_–CH_2_–)), 1.36–1.53 (m, 16H, (–C*H*_2_–C*H*_2_–C*H*_2_–P^+^–)), 2.01–2.11 (m, 8H, (–C*H*_2_–P^+^–)), 5.39–5.44 (dd, 1H, (C*H*_2_=CH–Ph–)), 5.90–5.97 (dd, 1H, (C*H*_2_=CH–Ph–)), 6.76–6.86 (dd, 1H, (CH_2_=C*H*–Ph–)), 7.57–7.80 (dd, 4H, (CH_2_=CH–*Ph*–SO_3_^−^)).

### 2.4. Synthesis of Poly(alkane-1,#-diylbis(tri-n-butylphosphonium) 4-vinylbenzenesulfonate) (PSSBP#) and Poly(tetrabutylphosphonium 4-vinylbenzenesulfonate) (PSSMP)

The poly(alkane-1,#-diylbis(tri-*n*-butylphosphonium) 4-vinylbenzenesulfonate) (PSSBP#) series and poly(tetrabutylphosphonium 4-vinylbenzenesulfonate) (PSSMP), which is a polymeric draw solute of the PSSBP# series, were synthesized via free radical polymerization. SSBP8 (12 g, 13.60 mmol) and AIBN (0.05 g, 2 mol% of SSBP8) were mixed into 20 mL of DMF and stirred for 24 h at 90 °C under a nitrogen atmosphere. The resulting polymer was dialyzed thoroughly against DMF and other impurities using a dialysis tube (molecular weight cut-off = 3.5 kDa) for 3 days. After evaporating the remaining distilled water, the product was dried in a freeze dryer for 24 h. Other members of the PSSBP# series, i.e., PSSBP6 and PSSBP4, were prepared using the same procedure and amounts of materials. PSSMP was synthesized using same procedure, except a different amount of AIBN (0.02 g, 1 mol% of SSMP) was used. 1H-NMR spectroscopy was used to identify and confirm the PSSBP# series and PSSMP, and the associated spectra are shown in Figure 3.

^1^H-NMR of PSSBP8 [400 Mhz, D_2_O, *δ*/ppm]: 0.73–0.88 (m, 18H, (C*H*_3_–CH_2_–)), 1.06–1.17 (m, 4H, (–C*H*_2_–CH–Ph–)), 1.18–1.49 (m, 38H, (–CH_2_–C*H*–Ph–), (CH_3_–C*H*_2_–C*H*_2_–CH_2_–P^+^–), (P^+^–CH_2_–(C*H*_2_)_6_–CH_2_–P^+^)), 1.89–2.16 (m, 16H, (–C*H*_2_–P^+^–)), 6.09–7.06 (s, 4H, (–CH_2_–CH–*Ph*–SO_3_^−^)), 7.34–7.93 (s, 4H, (–CH_2_–CH–*Ph*–SO_3_^−^)).

^1^H-NMR of PSSBP6 [400 Mhz, D_2_O, *δ*/ppm]: 0.80–0.87 (m, 18H, (C*H*_3_–CH_2_–)), 1.28–1.48 (m, 36H, (–C*H*_2_–CH–Ph–), (CH_3_–C*H*_2_–C*H*_2_–CH_2_–P^+^–), (P^+^–CH_2_–(C*H*_2_)_4_–CH_2_–P^+^)), 1.99–2.12 (m, 18H, (–CH_2_–C*H*–Ph–), (–C*H*_2_–P^+^–)), 6.16–7.02 (s, 4H, (–CH_2_–CH–*Ph*–SO_3_^−^)), 7.38–7.88 (s, 4H, (–CH_2_–CH–*Ph*–SO_3_^−^)).

^1^H-NMR of PSSBP4 [400 Mhz, D_2_O, *δ*/ppm]: 0.71–0.99 (m, 18H, (C*H*_3_–CH_2_–)), 1.23–1.53 (m, 32H, (–C*H*_2_–CH–Ph–), (CH_3_–C*H*_2_–C*H*_2_–CH_2_–P^+^–), (P^+^–CH_2_–(C*H*_2_)_2_–CH_2_–P^+^)), 1.62–1.80 (m, 2H, (–CH_2_–C*H*–Ph–)), 2.01–2.33 (m, 16H, (–C*H*_2_–P^+^–)), 6.15–6.97 (s, 4H, (–CH_2_–CH–*Ph*–SO_3_^−^)), 7.37–7.87 (s, 4H, (–CH_2_–CH–*Ph*–SO_3_^−^)).

^1^H-NMR of PSSMP [400 Mhz, D_2_O, *δ*/ppm]: 0.74–0.90 (m, 12H, (C*H*_3_–CH_2_–)), 1.27–1.50 (m, 18H, (–C*H*_2_–CH–Ph–), (CH_3_–C*H*_2_–C*H*_2_–CH_2_–P^+^–)), 1.97–2.14 (m, 9H, (–CH_2_–C*H*–Ph–), (–C*H*_2_–P^+^–)), 6.18–7.93 (s, 4H, (–CH_2_–CH–*Ph*–SO_3_^−^)).

### 2.5. FO Performance

Water flux is a key property of the FO process and was investigated herein. The water flux was measured using a small-scale FO system connected to custom-made, cap-fixed L-shaped glass tubes. A membrane [Hydration Technologies Inc. (HTI), thin film composite FO membrane] was placed in a channel (diameter: 2.3 cm) between two L-shaped glass tubes. Draw solutes were added to one tube facing the active layer of the membrane, and distilled water as a feed solution was added into the other tube. The glass tubes were placed under an air atmosphere at room temperature. Measurement of water flux was performed in the active layer facing draw solution (AL-DS) mode. Depending on the orientation of the FO membrane, the degree of internal concentration polarization (ICP) phenomenon, which is one of the factors that reduces water flux, is changed. Particularly, it is well known that AL-DS mode exhibits less ICP phenomenon than active layer facing feed solution (AL-FS) mode. Thus, we selected AL-DS mode [35,36,37,38,39,40,41,42].

The draw and feed solutions were stirred using a magnetic bar and a solenoid (AS ONE, OCTOPUS CS-4) simultaneously. Water flux was determined by measuring the height difference of the solution before and after FO. The water flux [*J_w_*, (L m^−2^ h^−1^, LMH)] was calculated from the volumetric change of the draw solution using Equation (1).

(1) $$J_{w}=\frac{\Delta V}{A\Delta t}$$

where ΔV is the volume change of the draw solution over time, Δ*t*, and *A* is the effective membrane area (4.15 × 10^−4^ m^2^).

The reverse solute flux (*J_s_*) was determined by analyzing the quantity of solute that diffused from the draw solution into the feed solution during FO and the amount of total dissolved solid (TDS) in the feed solution. The reverse solute flux (*J_s_*, g m^−2^ h^−1^, gMH) was calculated from the difference between the conductivity of the feed solution before and after FO using Equation (2).

(2) $$J_{s}=\frac{\Delta \left(\mathrm{CV}\right)}{A\Delta t}$$

where ΔC is the concentration change of the feed solution after time, Δ*t*, and ΔV is the volume change after time, Δ*t*.

The specific reverse solute flux (*J_s_*/*J_w_*, g L^−1^) indicates the leakage of draw solute to feed solution, which can be calculated by dividing reverse solute flux into water flux

## 3. Results and Discussion

### 3.1. Synthesis and Characterization of Poly(alkane-1,#-diylbis(tri-n-butylphosphonium) 4-vinylbenzene-sulfonate) (PSSBP#)

**Figure 2:**
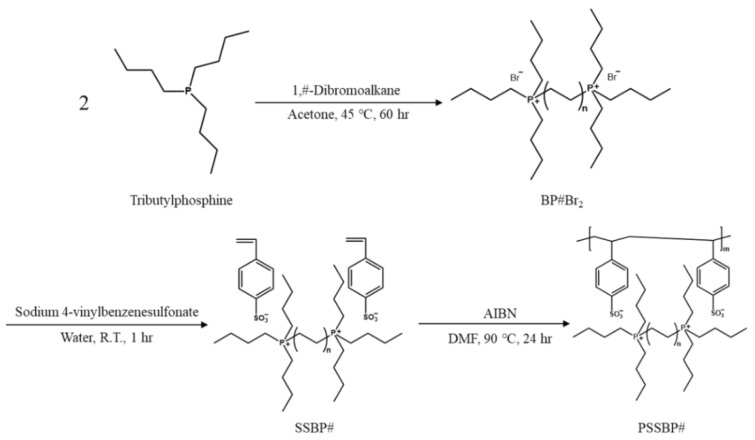
Preparative and synthetic scheme for dicationic phosphonium bromide (BP#Br_2_), where # is number of carbon atoms in centered bridge structure of dicationic phosphonium moiety, dicationic phosphonium 4-vinylbenzeneesulfonate (SSBP#), and poly(dicationic phosphonium 4-vinylbenzenesulfonate) (PSSBP#).

Figure 2 shows the overall synthetic scheme for the poly(alkane-1,#-diylbis(tri-*n*-butylphosphonium) 4-vinylbenzenesulfonate) (PSSBP#) polymer series. A series of dicationic phosphonium bromides, BP#Br_2_, constituting PSSBP# was prepared via Menshutkin SN_2_ reaction between tributylphosphine and the 1,#-dibromoalkane series. The Menshutkin SN_2_ reaction occurs when the unshared electron pair of tributylphosphine attacks both ends of alpha carbons bonded covalently to bromine atom of the 1,#-dibromoalkane series, which has a relatively low electron density. The BP#Br_2_ series was obtained by precipitation and purified by Soxhlet extraction, removing impurities such as the 1,#-dibromoalkane series, tributylphosphine, and (#-bromoalkyl) tributylphosphonium bromide. These impurities were moieties that were not fully reacted with both alpha carbons. The ^1^H-NMR spectra of the pure BP#Br_2_ series are shown in Figures S1–S3 (see Supplementary Materials). According to Figures S1–S3, the alkyl (*δ* = 0.89–0.99 [peak a)] and alkylene groups [*δ* = 1.31–1.81 (peak b), 2.11–2.33 (peak c)] were identified, and the integral ratio of each region was in agreement with the ratio of the predicted number of proton atoms. Based on these results, we concluded that the dicationic phosphonium bromide (BP#Br_2_) was successfully synthesized. The monomers, i.e., SSBP8, SSBP6, SSBP4, and SSMP, and the monomer from PSSMP as a referential polymer for PSSBP# polymers were obtained by solvent extraction using distilled water and dichloromethane. This procedure utilizes the partition coefficient of the solute between water as a polar solvent and dichloromethane as a nonpolar solvent. Thus, the resultant monomers, SSBP# series, and SSMP were dissolved in the nonpolar dichloromethane layer, which was removed to obtain the desired monomer series. Structural analysis of the corresponding pure monomers was performed by ^1^H-NMR spectroscopy (Figures S4–S7). The bromide of BP#Br_2_ and tetrabutylphosphonium bromide was substituted with 4-vinylbenzenesulfonate, as confirmed by the presence of vinyl [*δ* = 5.22–6.86 (peak d and e)] and aryl groups [*δ* = 7.38–7.79 (peak f)] whose peaks were unidentified in the ^1^H-NMR spectra shown in Figures S1–S3. In addition, the integral ratio of each peak was calculated and agreed well with the theoretical number of protons, indicating that anion exchange was successful, and the monomers were pure.

**Figure 3:**
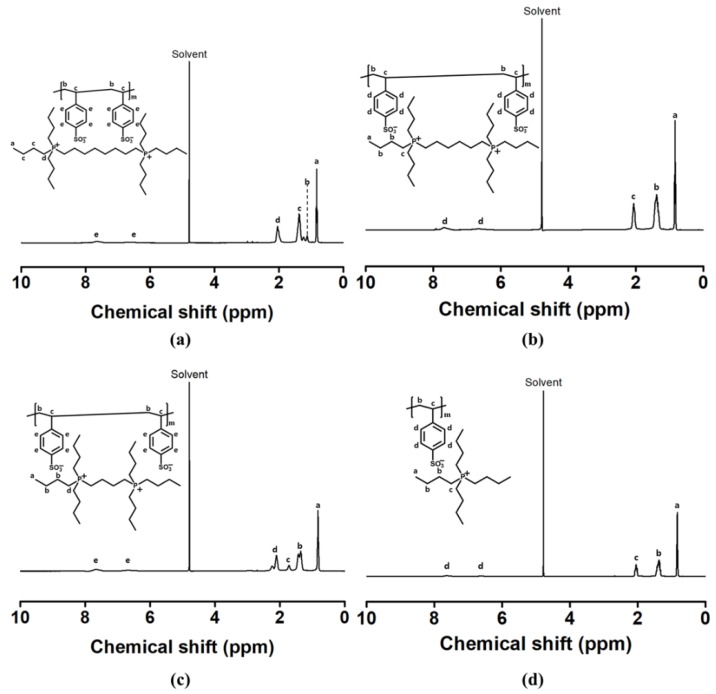
Proton nuclear magnetic resonance (^1^H-NMR) spectra: (**a**) polymer PSSBP8, (**b**) PSSBP6, (**c**) PSSBP4, and (**d**) PSSMP.

Finally, the PSSBP# series was polymerized via free radical polymerization in solution induced by the AIBN initiator. Structure confirmation for the PSSBP# series and PSSMP was achieved via ^1^H-NMR (Figure 3). According to Figures S4–S7 (see Supplementary Materials), in the chemical shift range of 5.20 to 6.89, the vinyl groups of the monomers constructing each polymer were identified. As the free radical polymerization proceeded, the peaks corresponding to the vinyl groups of each monomer disappeared, verifying that polymerization was successful.

### 3.2. Viscosity

**Figure 4:**
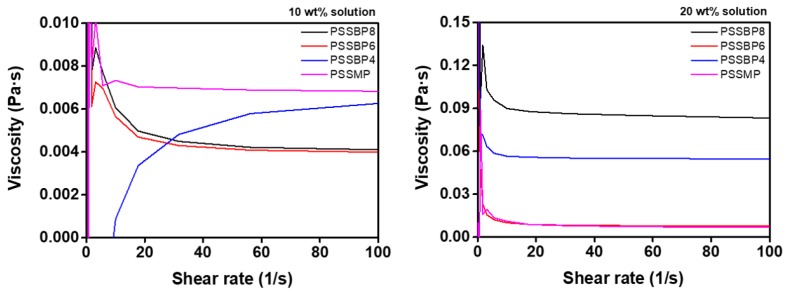
Viscosity of PSSBP# series and PSSMP according to shear rate at room temperature.

Viscosity significantly affects the FO behavior of draw solutions and the overall efficiency of the FO process. High viscosity of draw solutions indicates that the ionic diffusion coefficient of the draw solute is low [43]. This can induce internal concentration polarization (ICP) and external concentration polarization (ECP) in the membrane, resulting in decreased osmotic potential between the feed and draw solutions, which causes reduced water flux in FO [44,45,46]. Figure 4 shows the viscous behavior of the PSSBP# series and PSSMP aqueous solutions at different concentrations. The viscosities of the PSSBP# series and PSSMP were measured according to the shear rates, which ranged from 0.1 to 100 s^−1^ at room temperature. As expected, the viscosity values increased with increasing solute concentration (Figure 4), which was similar to those of other polymeric-type draw solutes [27,31,47]. Additionally, for the 10 and 20 wt% draw solutions, the viscosities of PSSBP8, PSSBP6, PSSBP4, and PSSMP solutions ranged between 0.004 and 0.007 Pa·s and between 0.007 and 0.080 Pa·s, respectively. The viscosity values of the PSSBP8, PSSBP6, PSSBP4, and PSSMP solutions are generally similar due to their similar molecular weight, indicating that the PSSBP# draw solutes are suitable for FO, which can be attributed to their ability to trigger minimal concentration polarization while maintaining low viscosities.

### 3.3. Conductivity

**Figure 5:**
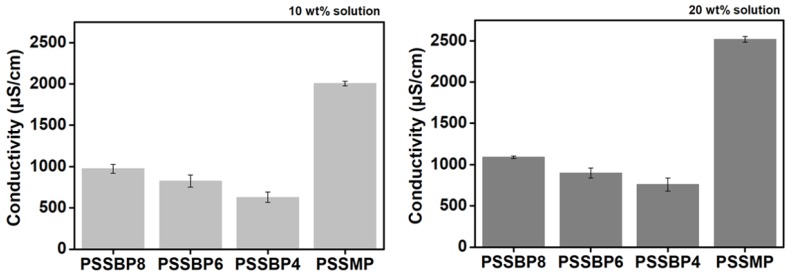
Conductivity of aqueous PSSBP# and PSSMP solutions according to solution concentration.

Conductivity is an indicator of the amount of draw solute ions in the draw solution and a measure of ion mobility. In addition, conductivity is affected by the dissociation degree of the ions, which is closely related to the osmotic pressure. Typically, higher conductivity leads to an increase in the osmotic pressure [48]. The conductivity of each draw solution was measured at solution concentrations of 10 and 20 wt% at room temperature, as shown in Figure 5. As the concentration of all draw solutions increased, the conductivity increased as expected due to the inherent properties of conductivity. The conductivity of the PSSMP draw solution was relatively higher than those of the PSSBP# draw solutions at all solution concentrations tested. This indicates that PSSMP will induce higher osmotic pressure than those of the PSSBP# series, which exhibit better FO properties when PSSMP is used as a draw solution. This is likely due to the structural differences of the cations constituting each polymer. Interestingly, at all solution concentrations, the conductivities of the PSSBP# solutions decreased as the number of carbons in the central bridge structure of the dicationic phosphonium decreased. This is because the flexibility of each diphosphonium increases as the length of the alkylene constituting the central bridge structure of the dicationic moiety is increased from # = 4 to # = 8 [49]. For further discussion, electrical conductivity of polyelectrolyte solution was quantitatively considered based on the counterion condensation model by Manning et al. [50,51]. One of the crucial effects for explaining this theory is the charge-solvent effect, which occurs by screening hydrodynamic interaction between chain segments of the polyion [50]. As the number of central carbons in the counterion (diphosphonium) constituting PSSBP# decreases, the charge density of each dication increases. In the case of the PSSBP# series, the charge density of the counterion of PSSBP4 will be the highest, and this will make it easier to interact with the polyion than those of PSSBP6 and PSSBP8. This phenomenon causes screening of hydrodynamic interaction between polyion segments and consequently leads to a decline in the electroconductivity of an aqueous solution. Therefore, the conductivity of the PSSBP# series shows the tendency shown in Figure 5. 

### 3.4. Osmotic Pressure

Osmosis is the spontaneous diffusion of water through a membrane, from the feed solution with a lower osmotic potential to the draw solution with a higher osmotic potential in FO [52]. Therefore, high diffusion of the draw solute in an aqueous state is necessary for the water molecules of the influent water to actively diffuse towards the draw solution [53]. Osmotic pressure is an indicator of FO performance that can be easily mentioned below and is a function of the solution concentration, which can be explained by the Van’t Hoff equation [Equation (3)] [54].

(3) $$\Pi =C_{i}RT$$

where *Π* is the osmotic pressure, *C_i_* is the molar concentration of solute *i* in a dilute solution, *R* is the gas constant, and *T* is the absolute temperature.

**Figure 6:**
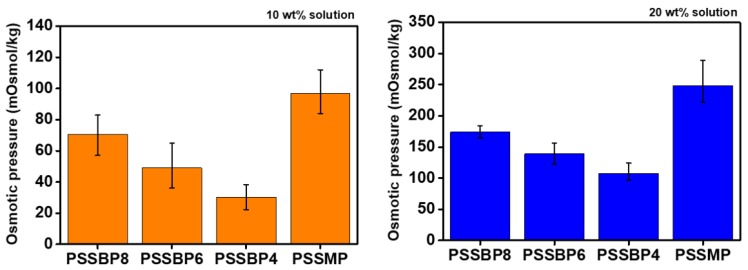
Osmotic pressure of the PSSBP# series and the PSSMP aqueous solutions according to solution concentration by cryoscopic method.

The cryoscopic method was used to measure osmotic pressure to investigate the applicability of the PSSBP# series as draw solutes. According to Figure 6, the osmotic pressures of PSSBP8, PSSBP6, PSSBP4, and PSSMP were approximately 70.5, 49.0, 30.3, and 97.0 mOsmol/kg, respectively, at a 10 wt% draw solution. Additionally, for the 20 wt% draw solution, the osmotic pressures of the PSSBP# series and PSSMP were approximately 174.0, 139.0, 107.7, and 248.5 mOsmol/kg, respectively. These results indicate that the osmotic pressure increases as the concentration of the aqueous solution increases. This result is experimental proof of the validity of the Van’t Hoff equation [Equation (3)] and shows the same behavior as other polyelectrolyte systems [55]. Interestingly, in Figure 6, the osmotic pressure of PSSMP was typically higher than those observed in the PSSBP# series.

According to the Stoke-Einstein equation, the diffusion coefficient of a particle can be expressed by Equation (4) [56].

(4) $$D=\frac{kT}{6\pi \eta r}$$

where *D* is the diffusion coefficient, *k* is the Boltzmann constant, *T* is the absolute temperature, *η* is the viscosity, and *r* is the radius of the spherical particle.

The osmotic pressure in an aqueous solution containing nonionic or ionic materials is closely related to the diffusion of the materials [57]. Higher degrees of diffusion result in more facile hydration by water molecules from the opposite side of the membrane [58]. For dicationic and monocationic systems, when each polymer, e.g., the PSSBP# series and PSSMP, was dissociated by water molecules, and the polymeric cations and anions were mobilized in an aqueous state, the osmotic pressure of the polymer solution was determined by the diffusion coefficient of the ions. As can be seen from structures of the PSSBP# series and PSSMP, the dicationic phosphonium and tetrabutylphosphonium cation can move relatively freely compared to the anion, which indicates that cation diffusion mostly controls osmotic potential. Consequentially, the osmotic pressure of dicationic and monocationic systems can be determined by evaluating the diffusion of each cation. Assuming the cations constituting each polymer are spherical, this result can be explained by particle size in the Stoke-Einstein equation. According to Figure 6, PSSMP exhibits a higher osmotic pressure than those of the PSSBP# series because the size of the tetrabutylphosphonium ion is relatively smaller than the other dicationic phosphoniums constituting PSSBP#.

### 3.5. Recovery Properties

**Figure 7:**
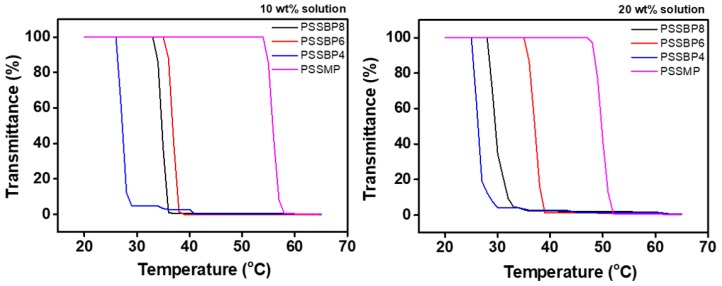
Transmittance curves of PSSBP# series and PSSMP aqueous solutions according to temperature variation.

The separation of draw solutes from the pure water and draw solution mixture during recovery in the FO process is essential for the recycling of draw solutes. Among the recovery methods of thermal-responsive draw solute, the LCST is the critical temperature at which, when the temperature is raised, the draw solute and water changes from homogeneous to heterogeneous phase. To measure the LCST of the PSSBP# series and PSSMP in an aqueous solution using UV-Vis spectrophotometry, the temperature of the phase change must be determined using a temperature versus transmittance curve. The monomers of the PSSBP# series, e.g., SSBP8, SSBP6, and SSBP4, showed LCST properties. However, the LCSTs of the monomers were too low to be measured at values close to 0 °C (data now shown). Consequently, we concluded that the monomers cannot be utilized as draw solutes in FO. Figure 7 displays temperature versus transmittance curves of the PSSBP# series and PSSMP aqueous solutions. Each LCST was similarly determined by obtaining transmittance curves using a UV-Vis spectrophotometer at 550 nm equipped with a temperature controller. The temperature versus transmittance curves showed a drastic change at each critical temperature. When the temperatures of the PSSBP# and PSSMP solutions were lower than the critical temperature, the transmittance values of each aqueous solution were almost 100%, indicating good solubility of draw solutes. Conversely, when the temperature of the solutions was higher than the critical temperature, transmittance was approximately 0%. This indicates that the solutions became heterogeneous, suggesting that PSSBP# and PSSMP were separated from the water phase. Furthermore, as the concentration of the aqueous solutions increased, the LCST decreased. For instance, when the concentration of aqueous solution was 10 wt%, the LCSTs of PSSBP8, PSSBP6, PSSBP4, and PSSMP were 33, 39, 27, and 55 °C, respectively. When the concentration was increased to 20 wt%, the LCSTs decreased to 30, 38, 26, and 50 °C, respectively. When the temperature of the aqueous polymer solution was lower than the LCST, the interactions between ions, e.g., dicationic phosphonium and sulfonate anions, and water molecules were more dominant than those of the ion-ion interactions between the dicationic phosphonium and sulfonate anions. However, as the temperature increased, ion-ion interactions became more influential compared to those of the ion-water interactions, resulting in polymer aggregation and conversion of the aqueous polymer solution to a heterogeneous phase. According to Wu et al., in detail, the coil-to-globule transition that occurs when heat is applied above a certain temperature determines the intrachain structure of thermal-responsive polymers, coil or globule form, due to thermodynamic stability of the polymers generated during the process, which is determined by polymer chain density distribution [59]. As shown in Figure 7, when thermal energy is applied to the aqueous PSSBP# solution, a physicochemical interaction between polymer main chain and each diphosphonium leads to the transition from coil to globule form. The critical temperature, LCST, is determined by thermodynamic stability of the aqueous PSSBP# solutions. Taking PSSBP4 as an example, this polyelectrolyte can be thermodynamically stable with relatively little thermal energy compared to other PSSBP# series. We concluded that each LCST of a draw solute having dication, i.e., PSSBP#, is the result of structural differences of dications leading dissimilar polymer chain density. Moreover, due to the proximity of the LCSTs to room temperature, the PSSBP# series can be separated from the pure water and draw solution mixture with minimal energy (especially compared to that of the PSSMP system), which is advantageous for recovery.

### 3.6. Water and Reverse Solute Fluxes

**Figure 8:**
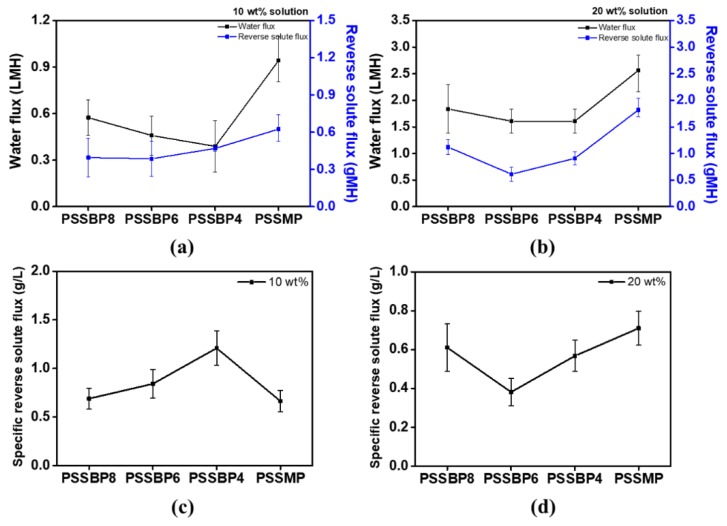
Water flux and reverse solute flux of PSSBP# series and PSSMP at solution concentration, (**a**) 10 wt% and (**b**) 20 wt% in room temperature in the FO process and specific reverse solute flux of the draw solutes at (**c**) 10 wt% and (**d**) 20 wt%.

To optimize the FO process, the bidirectional diffusion of water and draw solute must be considered [52]. Thus, the water and reverse solute fluxes were measured at various concentrations of each draw solute, i.e., the PSSBP# series and PSSMP, to determine the feasibility of PSSBP# as a draw solute. Measurements of water and reverse solute fluxes were performed at room temperature, which is lower than the LCSTs of all polymers. As expected from the previous osmotic pressure results (Figure 5), the water flux of each FO process increased with increasing concentration of all draw solutions from 10 to 20 wt% (Figure 8). When the concentration of PSSBP8, PSSBP6, PSSBP4, and PSSMP draw solutions was 10 wt%, water flux was 0.57, 0.46, 0.39, and 0.94 LMH, respectively, and increased to 1.84, 1.61, 1.61, and 2.56 LMH, respectively, for the 20 wt% draw solutions. The reverse solute flux was calculated by comparing the TDS of the feed solution before and after FO. Reverse solute flux is generated by mass transfer caused by back diffusion of the draw solute induced by size differences between the membrane pore and that of the draw solute and concentration differences between the feed and draw solutions. Therefore, as the difference in solution concentration increased, the reverse solute flux also increased. The reverse solute fluxes were 0.40, 0.39, 0.47, and 0.63 gMH for 10 wt% draw solutions of PSSBP8, PSSBP6, PSSBP4, and PSSMP, respectively. The respective 20 wt% draw solutions exhibited reverse solute fluxes of 1.12, 0.61, 0.91, and 1.82 gMH. At all concentrations, reverse solute flux was relatively higher than those of other draw solutes when PSSMP was used for FO. This is because the tetrabutylphosphonium cation is relatively less bulky compared to the dicationic phosphonium series and exhibits a relatively high mobility of the monocation. Because the size of tetrabutylphosphonium is relatively smaller than those of the dicationic phosphonium series, the reverse solute flux of the PSSMP system is higher than those observed for the PSSBP# series. Additionally, we also calculated specific reverse solute flux, which indicates the amount of draw solute outflow in the direction of feed solution—leakage of draw solute—during the FO process. Based on Figure 8c,d and other results, it can be concluded that the dicationic system showed superior FO performance to that of the monocationic system. For checking the level of PSSBP# series in FO performance perspective, we made a comparison between the FO performance of PSSBP# and that of conventional draw solute, sodium chloride. As a result of comparison, when 20 wt% sodium chloride aqueous solution was used as a draw solute, it was shown that higher electroconductivity (196.2 mS/cm), osmotic pressure (579.1 Osmol/kg), and water flux (22.97 LMH) were performed. However, after the FO process, reverse solute flux was calculated as over 10 gMH. This result signifies that sodium chloride was diffused through the membrane toward feed solution, which was significantly higher than that of PSSBP# series. Apart from desalination technology, we identified the oil-separating performance using the corresponding draw solute series to confirm wide scalability of industrial applications. The feed solution was mixed with olive oil and water at a ratio of 1:19, and emulsion was prepared using a homogenizer. The 20 wt% PSSBP# solution was selected as a draw solute. After the oil separation process run, water flux was found to be about 0.61 LMH. The novel and next-generation polymeric draw solute, PSSBP# series, is expected to be applied in various industrial fields such as oil separation and rare metal recovery from industrial wastewater.

## 4. Conclusions

A draw solute series with dumbbell-shaped dicationic structures, PSSBP#, where # is the number of carbon atoms in the central bridge structure of dicationic phosphonium moiety, was synthesized via free radical polymerization to examine its feasibility for use in FO. In aqueous solutions, the PSSBP# series showed LCSTs, which is essential for recovery in FO. The LCSTs of PSSBP8, PSSBP6, and PSSBP4 were approximately 30, 38, and 26 °C, respectively. The respective water fluxes were identified in AL-DS mode as 1.84, 1.61, and 1.61 LMH at 20 wt%, and the respective reverse solute fluxes were 1.12, 0.61, and 0.91 gMH the under same conditions. PSSBP#, a thermal-responsive homopolymer series containing a dumbbell-shaped dicationic phosphonium moiety, can surmount the typical trade-off relationship, which is a chronic problem of copolymer systems. Furthermore, this series exhibiting LCST characteristics has great potential in terms of draw solute recovery because its recovery temperature is close to room temperature, reducing the amount of energy required for recovery compared to those of another draw solute. This study provides inspiration in terms of structural modification for controlling recovery temperature in FO and a novel methodology for developing draw solutes.

## Figures and Tables

**Table 1:** Overview of previous research about external-stimulus-responsive draw solutes.

Category	Stimulation Factor	Draw Solute	Osmotic Pressure/Concentration	Feed Solution	Water Flux	Ref.
Chemical stimulus responsiveness	Gas	NH_4_HCO_3_	4.80 MPa/1.1 M	0.5 M NaCl	3.4 LMH	[7]
	Salt	Potassium tannate	1135 mOsmol kg^−1/^100 mM	Deionized (DI) water	6 LMH	[13]
		Al_2_(SO_4_)_3_	– ^a)^	– ^a^^)^	– ^a^^)^	[14]
		MgSO_4_	– ^a^^)^/240,000 ppm	5,050 ppm NaCl	4.1 LMH	[15]
	pH	PDMAEMA	1208 mOsmol kg^−1/^0.3 g g^−1^	DI water	6.3 LMH	[16]
Physical stimulus responsiveness	Magnetic field	PAA-MNP	1500 mOsmol kg^−1/^0.05 M	DI water	7.4 LMH	[18]
		MNP-FHg	– ^a)^	2,000 ppm NaCl	10.2 LMH	[21]
	Electric field	HA-PVA	– ^a)^/– ^a)^	DI water	1.5 LMH	[23]
		AMPS-DMEAMA	– ^a)^/– ^a)^	2000 ppm NaCl	2.09 LMH	[24]
	Light	PNIPAM/Ag-Fe_3_O_4_	– ^a)/^– ^a)^	– ^a)^	– ^a)^	[25]
	Temperature	PSSS-PNIPAM	2.87 MPa/33 wt%	0.6 M NaCl	3.5 LMH	[29]
		PNIPAM-PSA	3.5 atm/14.28 wt%	DI water	0.35 LMH	[30]
		PBET	– ^a)^/20 wt%	DI water	3.22 LMH	[31]
		GE_m_B_n_	– ^a)^/56 wt%	0.6 M NaCl	4.81 LMH	[32]
		PSSP5	20.85 atm/20 wt%	DI water	14.5 LMH	[27]
		PSSBP8	174.0 mOsmol kg^−1^/20 wt%	DI water	1.84 LMH	This Study

^a)^ Unidentified values in each reference.

## Supplementary Materials

The following are available online at https://www.mdpi.com/2073-4360/11/3/571/s1, Figure S1: Proton nuclear magnetic resonance (^1^H-NMR) spectrum of octane-1,8-diylbis(tri-*n*-butylphosphonium) bromide (BP8Br_2_), Figure S2: ^1^H-NMR spectrum of hexane-1,6-diylbis(tri-*n*-butylphosphonium) bromide (BP6Br_2_), Figure S3: ^1^H-NMR spectrum of butane-1,4-diylbis(tri-*n*-butylphosphonium) bromide (BP4Br_2_), Figure S4: ^1^H-NMR spectrum of octane-1,8-diylbis(tri-*n*-butylphosphonium) 4-vinylbenzene-sulfonate (SSBP8), Figure S5: ^1^H-NMR spectrum of hexane-1,6-diylbis(tri-*n*-butylphosphonium) 4-vinylbenzene-sulfonate (SSBP6), Figure S6: ^1^H-NMR spectrum of hexane-1,4-diylbis(tri-*n*-butylphosphonium) 4-vinylbenzene-sulfonate (SSBP4), Figure S7: ^1^H-NMR spectrum of tetrabutylphosphonium 4-vinylbenzenesulfonate (SSMP).
