# Supplementary material for: Polymer Having Dicationic Structure in Dumbbell Shape for Forward Osmosis Process
Source: Polymers (Basel). 2019 Mar 26;11(3):571. doi: 10.3390/polym11030571 (PMC6473941; doi:10.3390/polym11030571)
Supplement: Supplementary file 1 [file polymers-11-00571-s001.pdf]

Polymers Having Dicationic Structure in Dumbbell Shape for Forward Osmosis Process

Taehyung Kim, Changha Ju, Chanhyuk Park, Hyo Kang*

Department of Chemical Engineering, Dong-A University,

37 Nakdong-Daero 550beon-gil, Saha-gu, Busan 49315, Republic of Korea

* Corresponding Author. (E–mail: hkang@dau.ac.kr, Phone: +82 51 200 7720, Fax: +82 51 200 7728)

Supplementary Information

Figure S1. Proton nuclear magnetic resonance (^{1}H-NMR) spectrum of octane-1,8-diylbis(tri-*n*-butylphosphonium) bromide ($BP8Br_2$).

Figure S2. ^{1}H-NMR spectrum of hexane-1,6-diylbis(tri-*n*-butylphosphonium) bromide ($BP6Br_2$).

Figure S3. ^{1}H-NMR spectrum of butane-1,4-diylbis(tri-*n*-butylphosphonium) bromide ($BP4Br_2$).

Figure S4. ^{1}H-NMR spectrum of octane-1,8-diylbis(tri-*n*-butylphosphonium) 4-vinylbenzene-sulfonate (SSBP8).

Figure S5. ^{1}H-NMR spectrum of hexane-1,6-diylbis(tri-*n*-butylphosphonium) 4-vinylbenzene-sulfonate (SSBP6).

Figure S6. ^{1}H-NMR spectrum of hexane-1,4-diylbis(tri-*n*-butylphosphonium) 4-vinylbenzene-sulfonate (SSBP4).

Figure S7. ^{1}H-NMR spectrum of tetrabutylphosphonium 4-vinylbenzenesulfonate (SSMP).

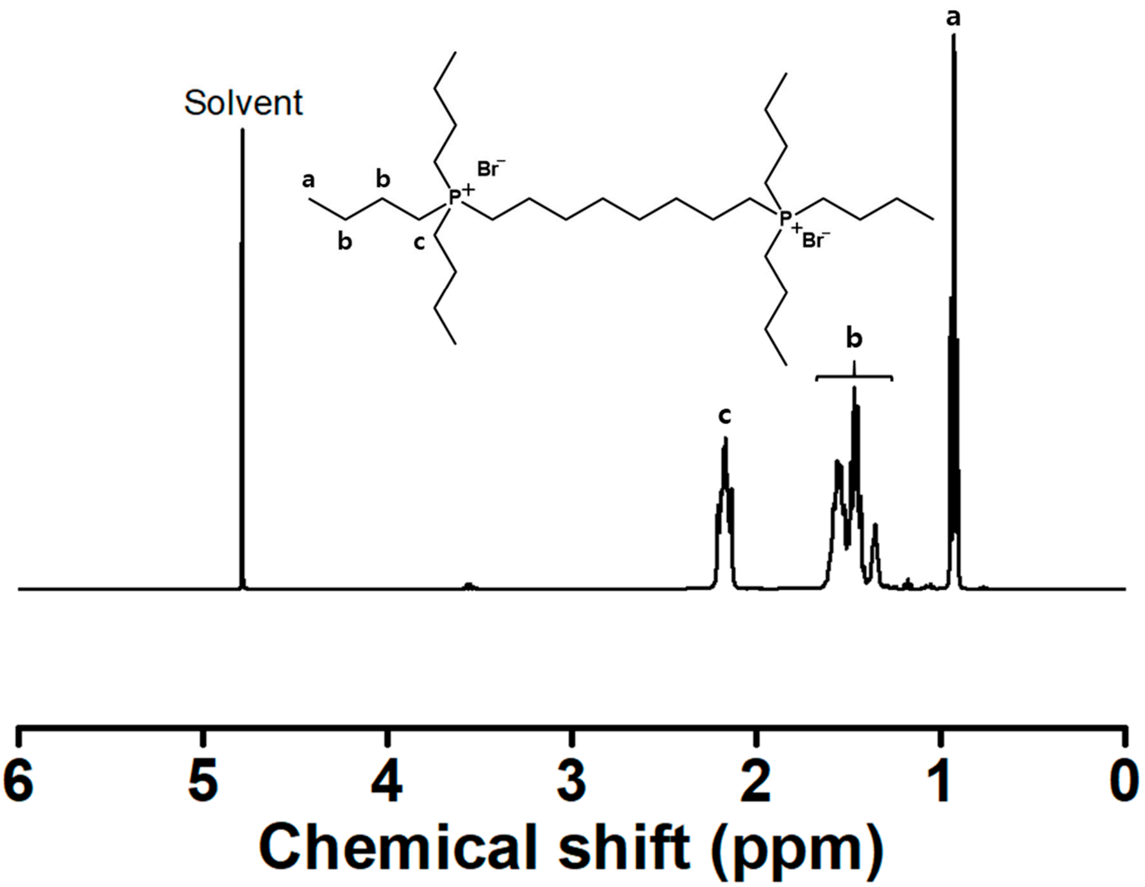

Figure S1. Proton nuclear magnetic resonance (^{1}H-NMR) spectrum of octane-1,8-diylbis(tri-n-butylphosphonium) bromide ($BP8Br_2$).

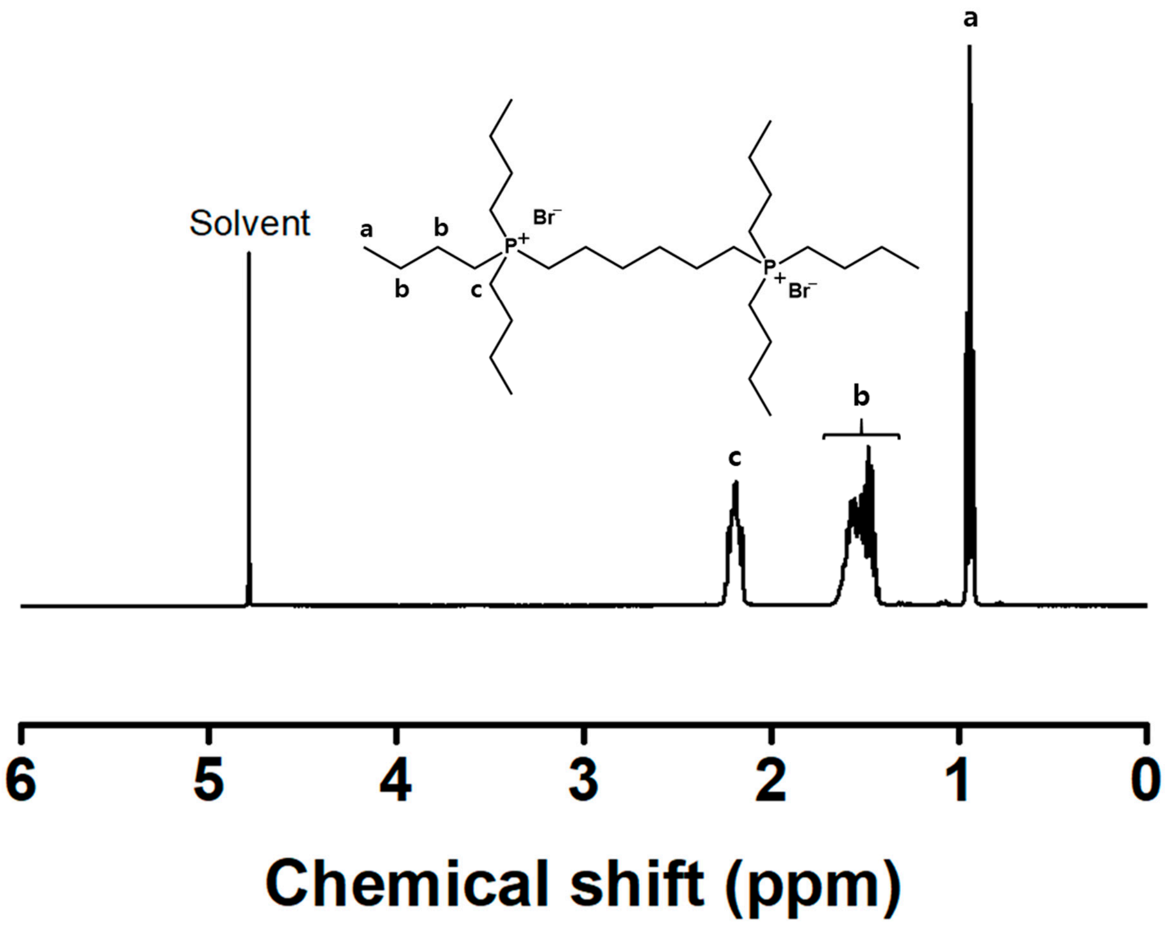

Figure S2. ^{1}H-NMR spectrum of hexane-1,6-diylbis(tri-*n*-butylphosphonium) bromide ($BP6Br_2$).

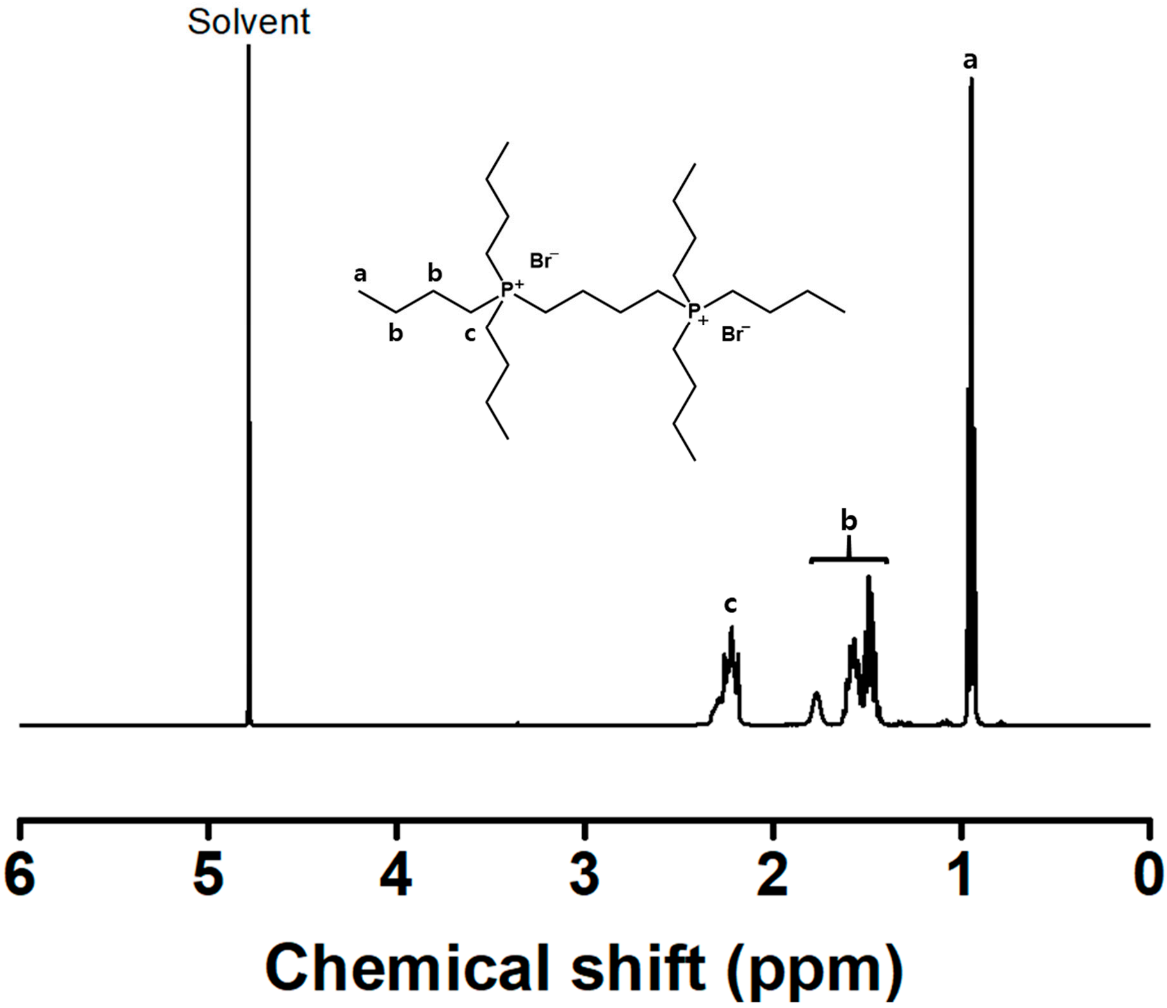

Figure S3. ^{1}H-NMR spectrum of butane-1,4-diylbis(tri-*n*-butylphosphonium) bromide ($BP4Br_2$).

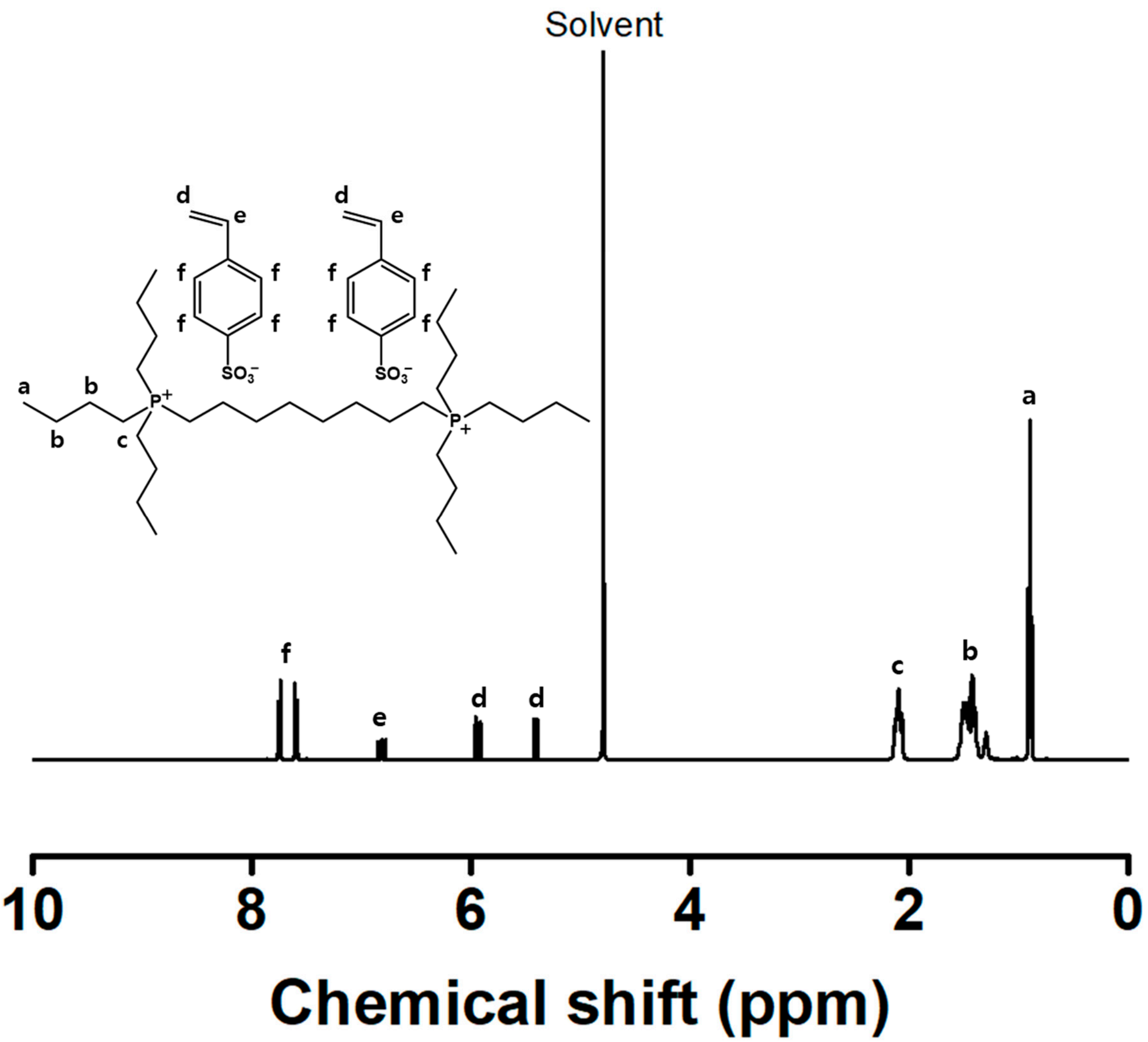

Figure S4. ^{1}H-NMR spectrum of octane-1,8-diylbis(tri-*n*-butylphosphonium) 4-vinylbenzene-sulfonate (SSBP8).

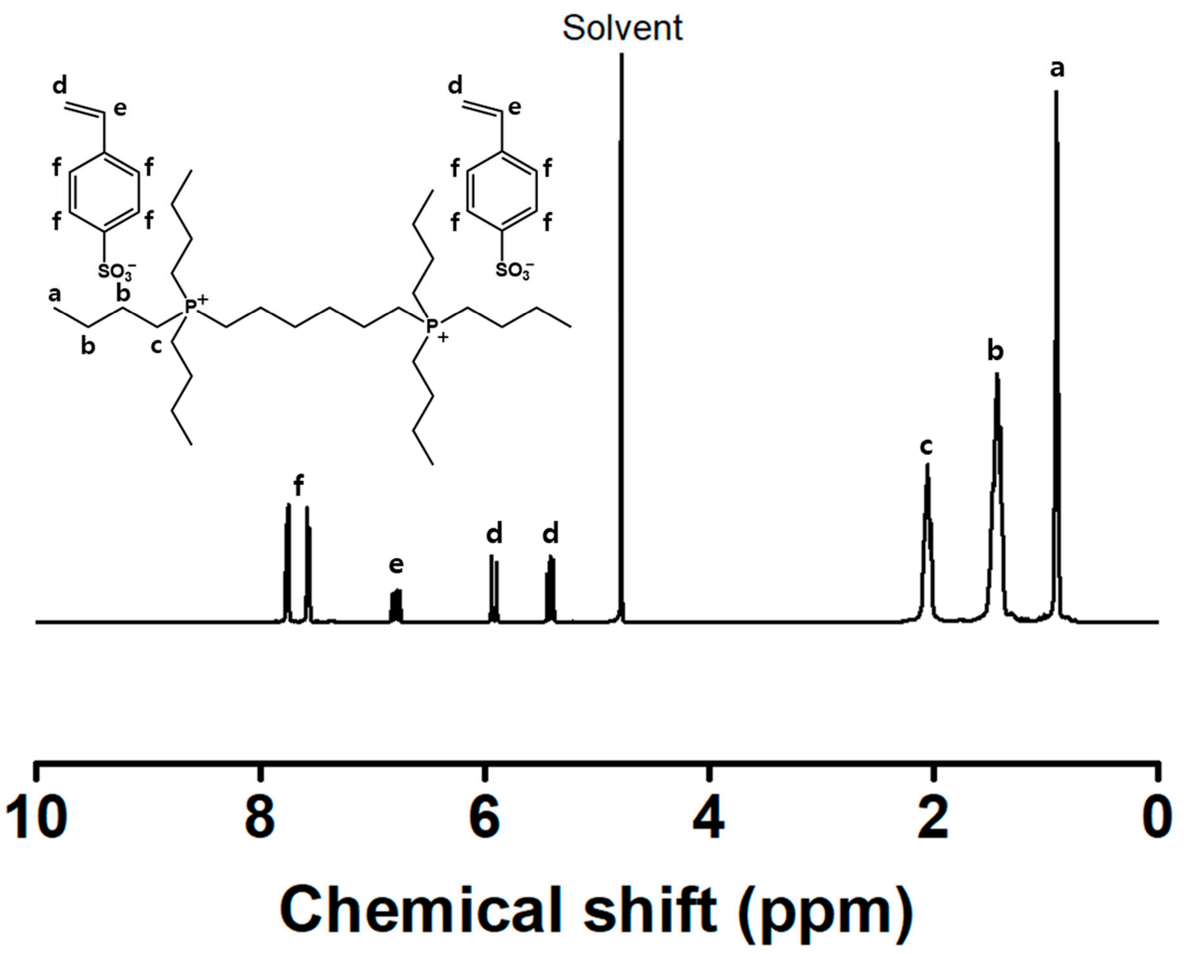

Figure S5. ^{1}H-NMR spectrum of hexane-1,6-diylbis(tri-*n*-butylphosphonium) 4-vinylbenzene-sulfonate (SSBP6).

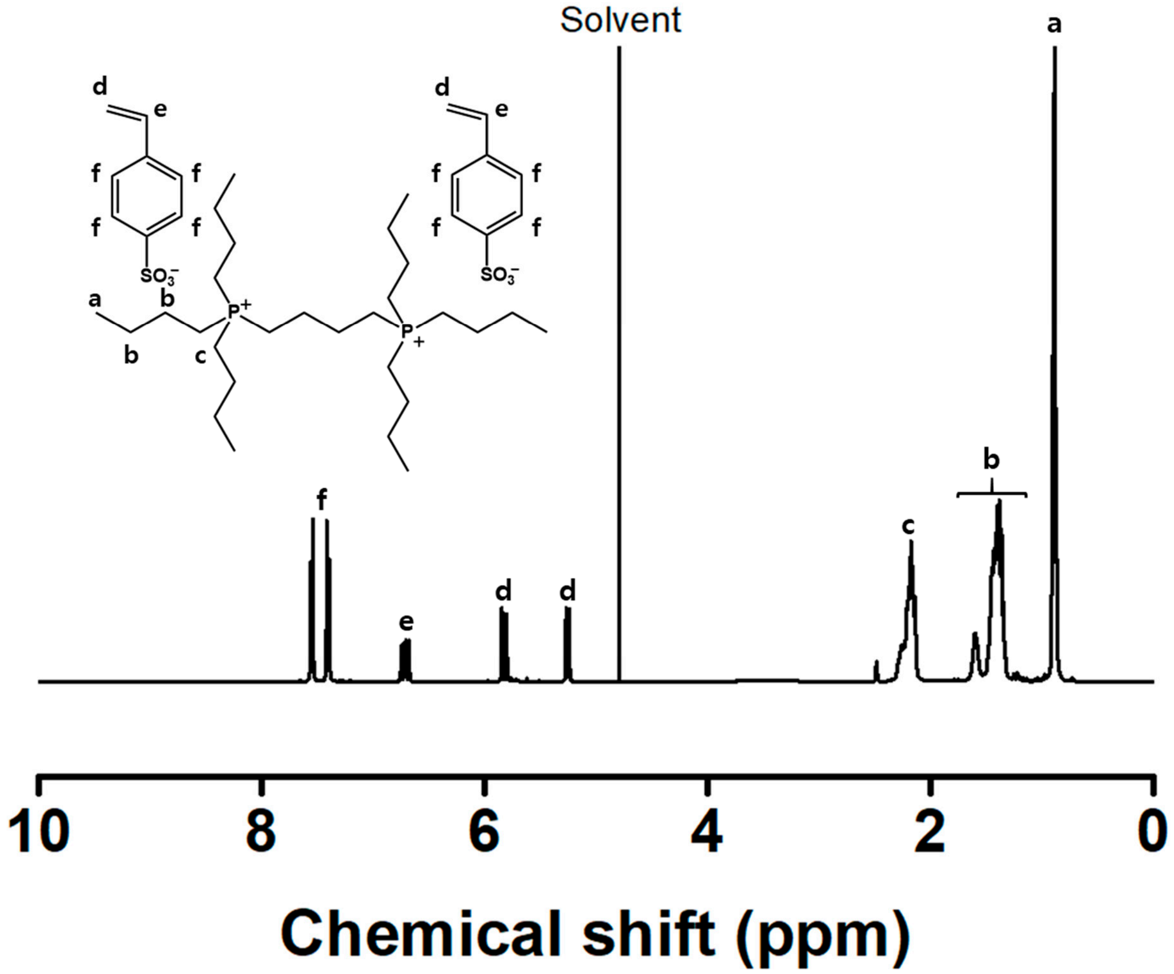

Figure S6. ^{1}H-NMR spectrum of butane-1,4-diylbis(tri-*n*-butylphosphonium) 4-vinylbenzene-sulfonate (SSBP4).

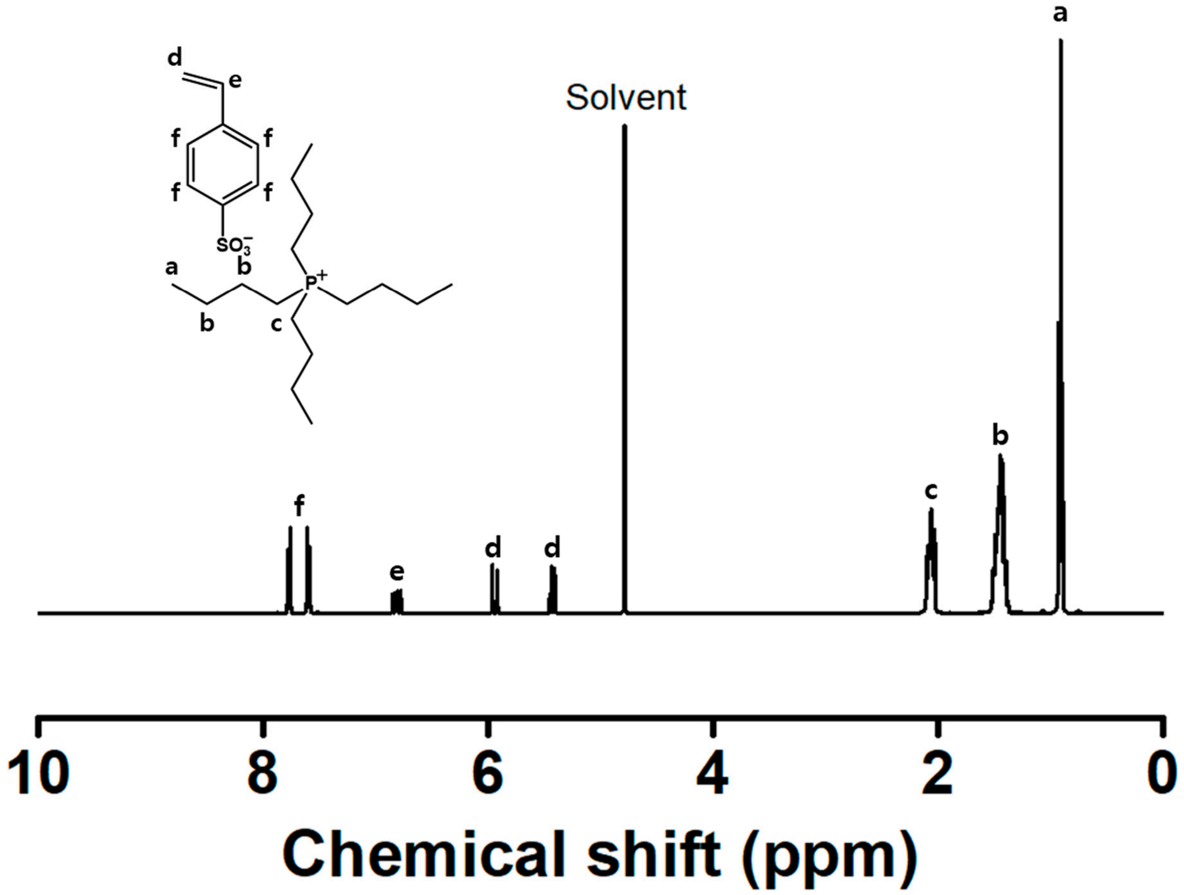

Figure S7. ^{1}H-NMR spectrum of tetrabutylphosphonium 4-vinylbenzenesulfonate (SSMP).
